# The Status of STAT3 and STAT5 in Human Breast Atypical Ductal Hyperplasia

**DOI:** 10.1371/journal.pone.0132214

**Published:** 2015-07-06

**Authors:** Aiping Shi, Jie Dong, Susan Hilsenbeck, Lirong Bi, Hong Zhang, Yi Li

**Affiliations:** 1 Department of Breast Surgery, The First Hospital of Jilin University, Changchun, Jilin, China; 2 Lester and Sue Smith Breast Center, Baylor College of Medicine, Houston, Texas, United States of America; 3 Department of Molecular and Cellular Biology, Baylor College of Medicine, Houston, Texas, United States of America; 4 Dan L Duncan Cancer Center, Baylor College of Medicine, Houston, Texas, United States of America; 5 Department of Molecular Virology and Microbiology, Baylor College of Medicine, Houston, Texas, United States of America; 6 Department of Pathology, MD Anderson Cancer Center, Houston, Texas, United States of America; University of Texas Health Science Center, UNITED STATES

## Abstract

Signal Transducer and Activation of Transcription factors (STAT3 and STAT5) play important roles in breast epithelial cell differentiation, proliferation, and apoptosis. They have been investigated extensively in established breast cancer, but their activation status in precancerous lesions has not been reported. Formalin-fixed, paraffin-embedded archival tissues from 59 cases of atypical ductal hyperplasia (ADH) and 31 cases of normal human breast tissue as well as 21 cases of usual ductal hyperplasias (UDH) were obtained from the First Hospital of Jilin University, China, and stained for pSTAT3 and pSTAT5 by immunohistochemistry. The median percentage of pSTAT5+ cells in ADH was 12%, not significantly deviant from that in normal breast. The median percentage of pSTAT3+ cells in ADH was 30%, significantly higher than that of normal breast. pSTAT3 and pSTAT5 were exclusive of each other—they were detected in different ADHs or in different cells within the same ADHs. In addition, both pSTAT3 and pSTAT5 were produced in similar percentages of cells in ADHs from cancer-free patients vs. ADHs that were adjacent to an invasive cancer. Our finding of a complementary expression pattern of pSTAT3 and pSTAT5 in ADH suggests that these two transcription factors may have feedback inhibitory effects on each other during early stages of breast cancer evolution, and that disruption of this inverse relationship may be important in the progression from early lesions to cancer, which exhibits positive association between pSTAT3 and pSTAT5.

## Introduction

Atypical ductal hyperplasia (ADH) is a frequently detected precancerous lesion in the breast after age 40–50—autopsy studies detected moderate to severe hyperplasia in over 30% of women aged 45–54 and ADH in 7% of women aged 20–54 [1–3]. ADH is a well-established precursor of breast cancer [4–7]. Women with ADH have approximately fivefold increased risk of developing breast cancer [6, 8–10]. In addition, gene expression profiling of ADH, ductal carcinoma in situ (DCIS), and invasive ductal carcinoma (IDC) showed that these three stages of breast cancer are highly similar to each other at the transcriptional level [11], further suggesting that ADH is a precursor during breast cancer evolution. However, very little is known about proteins and signaling pathways that are activated in these precancerous lesions. Knowledge of this aspect may help understand the evolution of these lesions to cancer and may help design prevention strategies to avert this progression.

The Signal Transducers and Activators of Transcription (STAT) family comprises seven transcriptional factors—STAT1, STAT2, STAT3, STAT4, STAT5a, STAT5b, and STAT6. They are made as latent protein in the cytoplasm [12], and are activated by phosphorylation on a tyrosine residue near the C-terminus, usually by a member of the JAK (Janus kinase) family, which is in turn activated by a ligand-bound receptor (such as cytokine receptors and the prolactin receptor). STATs may also be phosphorylated by other tyrosine kinases through growth factor signaling, as well as by Src [13]. After phosphorylation, STATs form homodimers as well as heterodimers through reciprocal phosphotyrosine-SH2 domain interactions, translocate to the nucleus, and activate their targets. STAT5 and STAT3 are two key STAT family members involved in mammary gland development and tumorigenesis [14]. pSTAT5 is detected in a subset of mammary epithelial cells, varying in percentage of cells during the menstrual cycle and reaching nearly 100% during late pregnancy and lactation [15–17]. Activated STAT5 regulates cell survival, proliferation, and differentiation into alveoli [12, 18–20]. pSTAT5 is rapidly deactivated at the onset of involution [16, 21]. STAT5 has been shown to be involved in human breast cancer—though not mutated, pSTAT5 can be detected in 20–70% of breast cancers depending on the cohort of cancer samples [17, 22, 23]. pSTAT5 is found primarily in ER+ tumors, but also in a subset of HER2+ (i.e., ErbB2+) tumors as well as tumors lacking ER, PR, and HER2 (triple-negative) [17, 22, 23]. pSTAT5 can also be detected in a subset of DCIS [3, 22, 24, 25] as well as in histologically benign breast epithelia adjacent to breast tumors [24]. In preclinical studies using mouse models and in correlative studies using human DCISs and tumor-adjacent epithelia, we have found that STAT5 suppresses apoptosis in mammary early lesions, promotes progression to cancer, and may be a valuable target for chemoprevention in women with increased risk of breast cancer [24]. On the other hand, STAT3 is the dominant protein activated at involution to activate apoptosis to remove excess alveolar cells to return the mammary gland to a more dormant state similar to the pre-pregnancy stage [14]. However, STAT3 is also a critical player in breast cancer [14, 20, 26, 27]. pSTAT3 is frequently detected in human breast cancer cell lines [28, 29] and tumor specimens including DCISs [30–34]. pSTAT3 and pSTAT5 have been reported to be positively associated in invasive breast cancers [30]. However, the status of pSTAT5 and pSTAT3 in early stages of breast cancer such as ADH has not yet been reported.

In the current study, we used immunohistochemistry to examine pSTAT5 and pSTAT3 in 59 ADHs and for comparison 31 normal breast tissue and 21 UDH samples. We also attempted to identify association between pSTAT5 and pSTAT3, and between these two STAT proteins and proliferation or apoptosis.

## Materials and Methods

### Tissue selection and preparation

All archival tissue specimens were obtained from patients with surgeries between 2010 and 2014 (Table 1) at the First Hospital of Jilin University, China. First Hospital ethics committee-approved written informed consent was obtained for the use of these samples in research from all patients or the next of kin. Immediately following surgical dissection, all tissues were fixed in formalin for overnight. The resulting paraffin-embedded tissue blocks were used for preparing 3-μm sections. Hematoxylin and eosin (H&E)-stained slides were evaluated by Dr. Lirong Bi at the First Hospital of Jilin University, and tissue specimens that demonstrated one or more pathological lesions (UDH and ADH) were selected.

**Table 1:** Characteristics of breast specimens.

Epidemiological characteristics	Normal breast tissues		Pure ADH		Tumor-adjacent ADH		UDH	
	Mean	SD	Mean	SD	Mean	SD	Mean	SD
Number of samples	31		31		28		21	
Age at diagnosis (yrs)	36.6	10.6	45.9	11.1	50.2	10.9	45.1	9.5
Age at last pregnancy (yrs)	26.7	8.1	28.8	8.7	29.9	7.9	27.5	9.5
No. of full-term pregnancies	2.3	1.1	2.2	1.3	2.4	1.2	2	1.3
ER+ cancer	N/A		N/A		26 of 28		N/A	
PR+ cancer	N/A		N/A		25 of 28		N/A	
Her2+ cancer	N/A		N/A		2 of 28		N/A	

Pure ADH (n = 31) and UDH (n = 21) tissues were biopsies or surgical resection specimens from noncancerous breasts (i.e., without DCIS or IDC). Additional ADH tissues (n = 28) were part of surgical specimens from patients diagnosed with breast cancer and were therefore designated as tumor-adjacent ADH. Normal breast tissues (n = 31) were obtained from healthy women with a single fibroadenoma and without any other detectable abnormalities. De-identified use of samples in this study was reviewed by the Institutional Review Board at Baylor College of Medicine and determined to represent use of Human Materials but not Human Subjects.

### Immunohistochemistry and TUNEL assay

Immunostaining was performed on de-paraffined tissues sections after antigen retrieval (simmering in 0.1M citric acid, pH6.0, at 145°C for 10 minutes in a pressure cooker). The Vectastain Elite ABC system (Vector Laboratories, Burlingame, CA) was used following manufacturer’s instructions. The antibodies used were anti-pSTAT5 (9359, 1:500, Cell Signaling), anti-pSTAT3 (9145, 1:500, Cell Signaling), and anti-Ki67 (2011–11, 1:500, Novocastra), and were incubated at 4°C overnight. Apoptotic cells were determined by the DeadEnd Fluorometric TUNEL System (Promega, Madison, WI). DAPI counterstain was used to visualize nuclei. For normal ducts and ADH-adjacent ducts, TUNEL-positive cells were scored in at least 10 high power (x40) fields per section, and at least 1000 cells were counted for each sample. All ADHs and UDHs were included for quantifying TUNEL-positive cells.

### Assessment of biomarkers by immunohistochemistry

The quality of phospho-epitope preservation in these paraffin-embedded tissues was controlled by staining one section per case for pHistone3. The few samples that did not show significant pHistone3 staining were excluded for further analysis. pSTAT5 and pSTAT3 staining were quantified by counting for nuclear-stained cells and were reported as percentage of positive cells with a duct or lesion. One section from each patient was used for each marker, and all ADHs and UDHs on a section were included for quantification. For baseline levels, at least 10 randomly selected normal ducts and 10 ADH-adjacent ducts were counted.

### Statistical analyses

Clinical characteristics were summarized with means and standard deviation for continuous variables and counts for categorical variables. Biomarker data were skewed in distribution and consequently pairwise Wilcoxon rank sum tests were used to compare groups and Wilcoxon signed rank tests were used to compare paired expression data (i.e. ADH and adjacent normal ducts). Biomarkers were summarized as medians. Associations between biomarkers were summarized with Spearman rank correlations, and displayed graphically as scatterplots and locally weighted smooth curves. P-values of 0.05 or less were considered significant and there was no adjustment for multiple comparisons. Analyses were accomplished using R version 3.1.3 (http://www.R-project.org/).

## Results

### The percentage of pSTAT3-positive cells, but not pSTAT5-postive cells, is increased in ADH compared to the normal breast tissue

**Fig 1:**
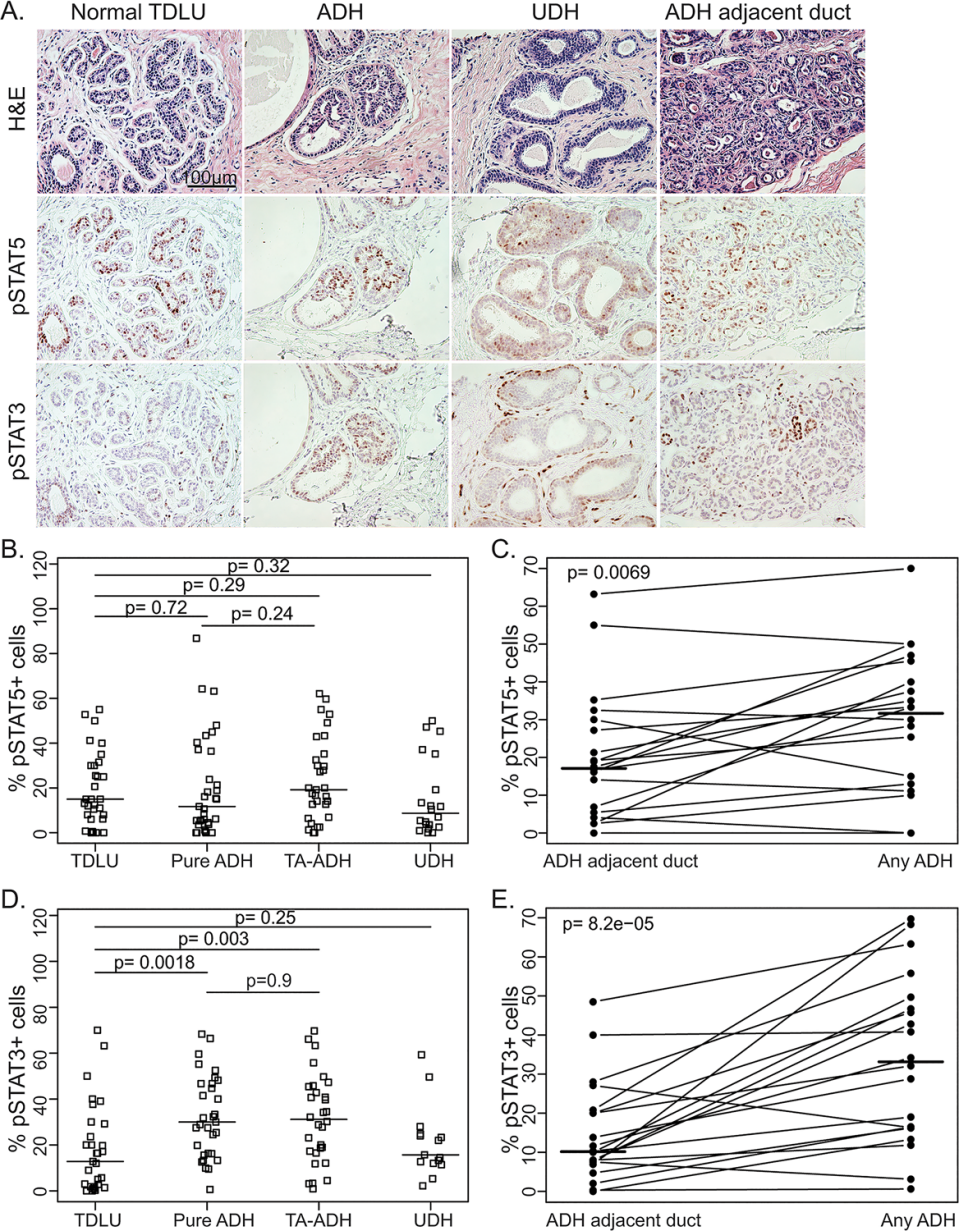
pSTAT5 and pSTAT3 status in human ADH. A. H&E staining (top panel), pSTAT5 (mid panel), and pSTAT3 IHC staining (bottom panel) of normal TDLU, ADH, UDH, and ADH-adjacent ducts. B. Quantification of pSTAT5 staining in normal TDLU, pure ADH, tumor-adjacent ADH (TA-ADH), and UDH, and pairwise comparisons shown by horizontal lines. C. Paired comparison for percentage of pSTAT5 positive cells in ADH and corresponding ADH-adjacent normal ducts. D. Quantification of pSTAT3 staining in normal TDLU, pure ADH, tumor-adjacent ADH (TA-ADH), and UDH, and pairwise comparisons shown by horizontal lines. E. Paired comparison for percentage of pSTAT3-positive cells in ADH and corresponding ADH-adjacent normal ducts.

We obtained from the First Hospital of Jilin University 59 ADHs (31 of which were from breasts without an invasive cancer while 28 of which were adjacent to an invasive cancer), and for comparison 31 normal breast tissues and 21 UDH samples. We stained them by immunohistochemistry for both pSTAT5 and pSTAT3. The median percentage of pSTAT5-positive cells was 15.0%, 11.7%, 19.2%, and 8.7% in normal TDLUs (terminal ductal lobular units), pure ADH, tumor-adjacent ADH, and UDH, respectively, and were not significantly different from each other (Fig 1A and 1B). However, among the 19 cases of ADH (including both types of ADH) that also had ADH-adjacent, histologically normal epithelia, paired comparison of pSTAT5 in ADH vs. normal ducts detected higher levels in ADH (31.65% vs. 17.10%, p = 0.0069, Fig 1C), which was similar to normal breast epithelia from benign breast.

The median percentages of pSTAT3-positive cells were 12.80%, 30%, 31.15%, and 15.7% in normal TDLU, pure ADH, tumor-adjacent ADH, and UDH, respectively. While the percentage of pSTAT3-positive cells in pure ADH was similar to that in tumor-adjacent ADH (p = 0.9, Fig 1D), the pSTAT3-postive cell frequency in either pure ADH or tumor-adjacent ADH was significantly increased compared to that in normal breast epithelia (p = 0.0018 and p = 0.003 respectively, Fig 1D). Furthermore, paired analysis also detected a significant increase of pSTAT3 in ADH over ADH-adjacent, histologically normal breast epithelia (33.15% vs. 10.15%, p = 8.2e-05, Fig 1E). There was no significant difference between UDH and normal TDLU (p = 0.25, Fig 1D). These observations suggest that pSTAT3 may play a role in progression to ADH and may serve as a progression marker during early stages of breast cancer evolution. In addition, these observations also suggest that pure ADH and tumor-adjacent ADH were similar in their regulation of pSTAT3 and pSTAT5; therefore, these two types of ADH were combined into one group for further studies.

### Complementary expression pattern of pSTAT5 and pSTAT3 in human ADH

**Fig 2:**
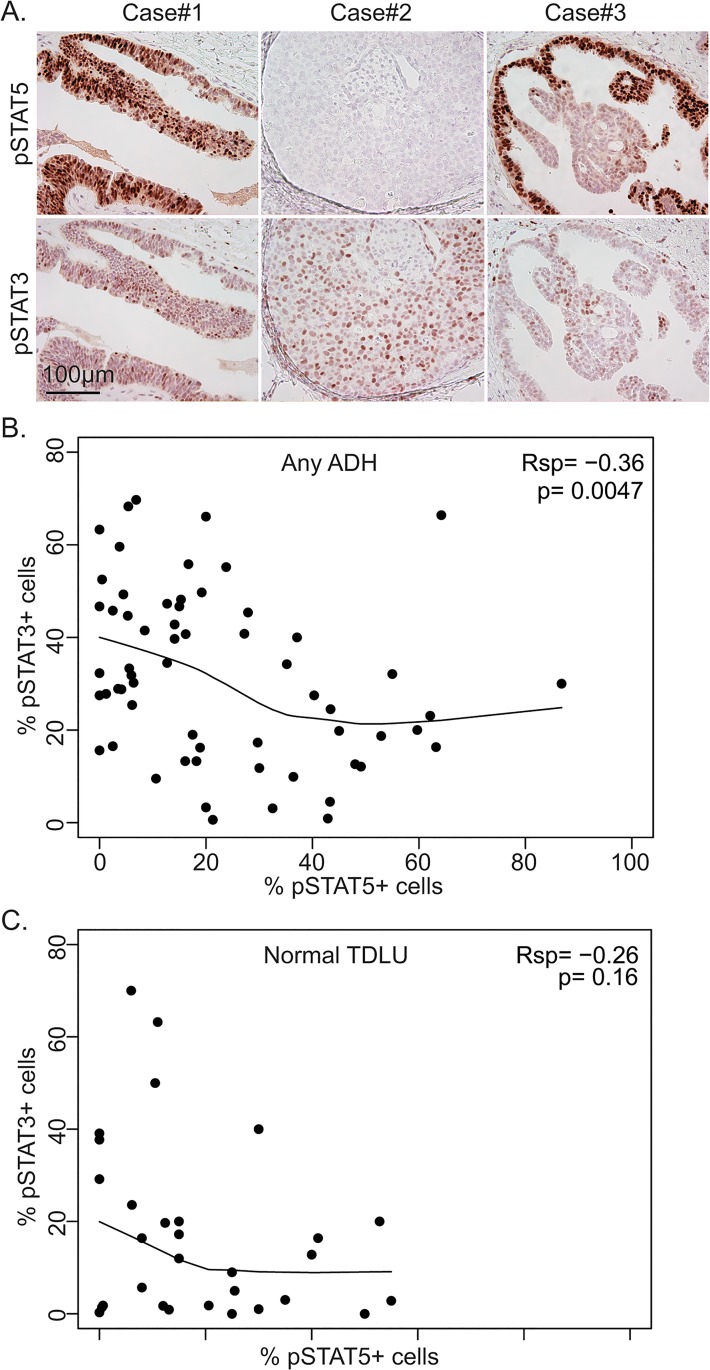
Complementary expression patterns of pSTAT5 and pSTAT3 in human ADH. A. Representative IHC staining for pSTAT5 (top panel) and pSTAT3 (bottom panel) in consecutive ADH lesions. B-C. Inverse correlation between percentage of pSTAT5+ and pSTAT3+ cells in ADH (B) or normal TDLU (C). Each dot represents an individual ADH lesion (B) or TDLU (C).

In normal breast epithelia, STAT5 and STAT3 are activated by different mechanisms and have different functions—STAT5 is activated during late pregnancy and lactation to promote alveologenesis and to maintain cell viability, while concurrent STAT5 deactivation and STAT3 activation at the onset of involution leads to alveolar cell apoptosis [14]. However, in invasive breast cancer, pSTAT3 has been reported to stimulate cell proliferation and to prevent apoptosis and is positively correlated with pSTAT5 [30]. In this relatively small set of normal breast samples, we did not detect a significant inverse relationship between pSTAT5-positive cells and pSTAT3-positive cells (p = 0.16, Rsp = -0.26, Fig 2C). However, among the 59 ADH cases, cases with higher pSTAT5-postive cells usually harbored fewer pSTAT3-positive cells, while cases with higher pSTAT3-positive cells often contained fewer pSTAT5-positive cells (p = 0.0047, Rsp = -0.36, Fig 2A and 2B). Careful examination of the spatial location of pSTAT5+ or pSTAT3+ cells within individual ADH cases revealed a complementary expression pattern as well—pSTAT5 and pSTAT3 were usually detected in different areas within a given precancerous lesion (Fig 2A, ADH#3). Taken together, these observations suggest that the coordinated control of pSTAT5 and pSTAT3 in normal breast epithelia is preserved in ADH and that pSTAT5 and pSTAT3 may have opposing effects on each other not only in normal breast epithelia but also in premalignant breast cells.

### pSTAT3 and pSTAT5 are not significantly associated with cell proliferation in ADH

**Fig 3:**
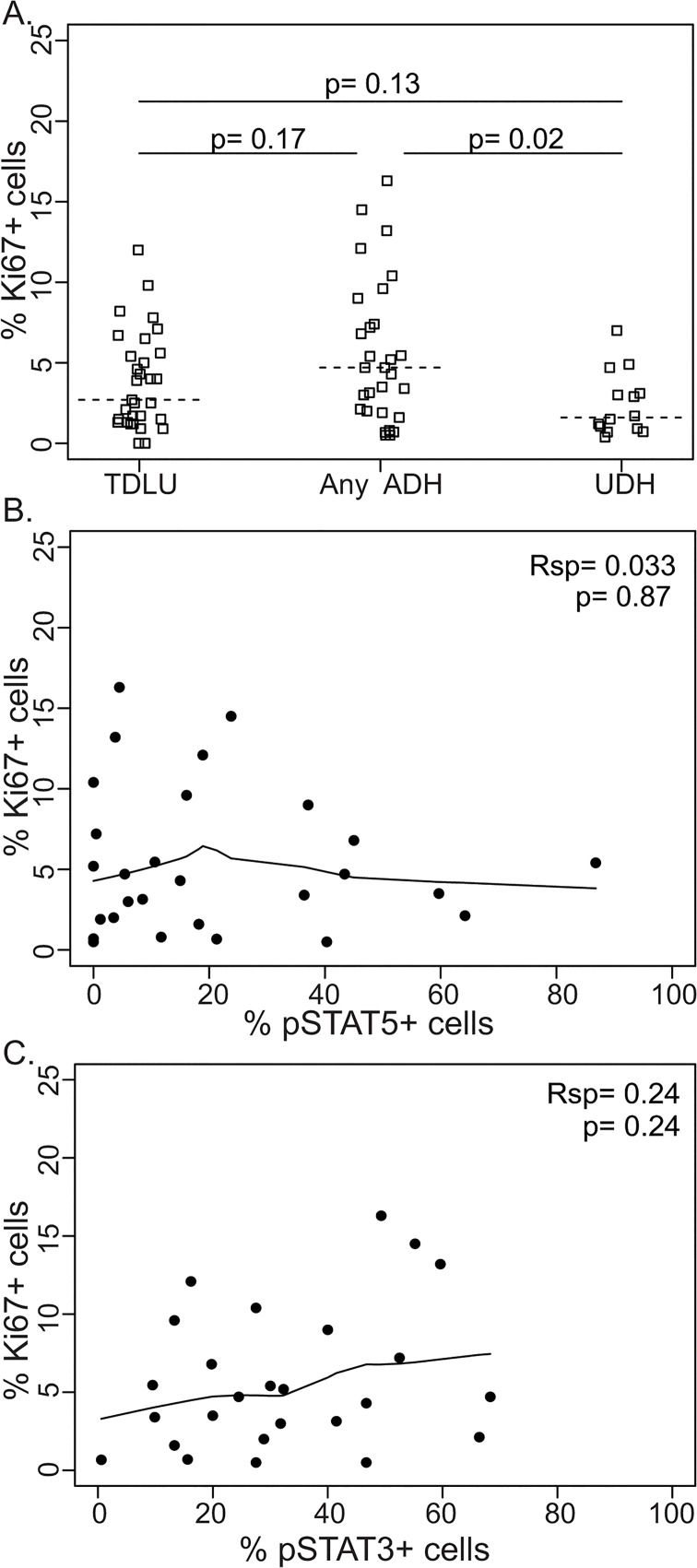
Cell proliferation rates in human ADH and their relationships with pSTAT5 and pSTAT3. A. Quantification of Ki67 staining in normal TDLU, ADH, and UDH. B-C. No association between percentage of pSTAT5+ (B) and Ki67+ cells or between pSTAT3+ (C) and Ki67+ cells in ADH. Each dot represents an individual ADH lesion.

We asked whether in these ADH samples, the rates of pSTAT5 or pSTAT3 were correlated with cell proliferation. The proliferation rate (as determined by Ki67 staining) in these ADH cases ranged from 0.5% to 16.3% with the median at 4.7% (n = 29), and was not significantly elevated compared to the value in normal breast tissues (median = 2.7%; n = 31; p = 0.17, Fig 3A). The median rate was similar to rates reported previously [5, 35–46]. The proliferation rate in UDH was 1.6% (n = 14), significantly lower than that in ADH (p = 0.02, Fig 3A). Neither pSTAT5-positive nor pSTAT3-positive cell percentages significantly correlated with proliferation (Fig 3B and 3C).

### pSTAT3 and pSTAT5 are not significantly associated with reduced apoptosis in ADH

**Fig 4:**
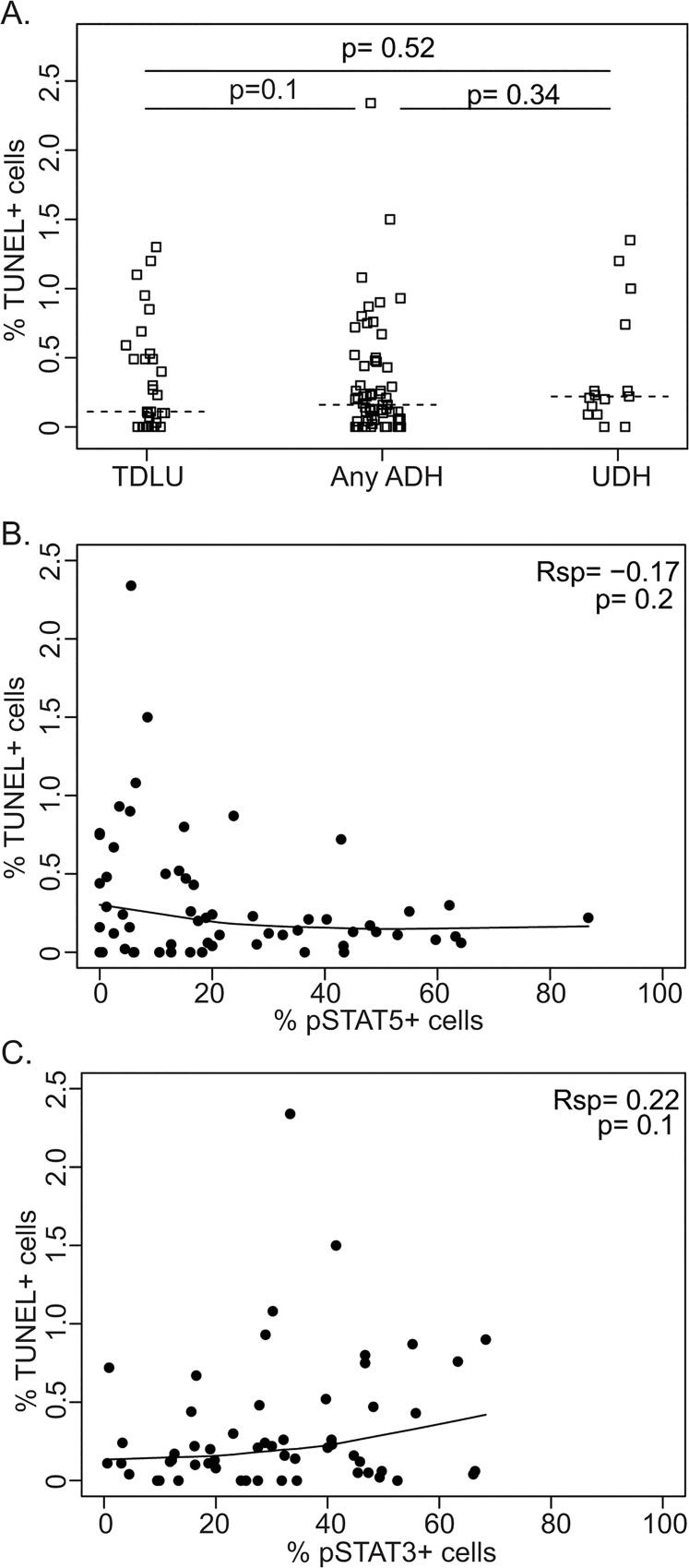
Cell apoptosis rates in human ADH and their relationships with pSTAT5 and pSTAT3. **A**. Quantification of TUNEL assay in normal ducts, ADH, and UDH. B-C. B-C. No association between percentages of pSTAT5+ (B) and TUNEL+ cells or between pSTAT3+ (C) and TUNEL+ cells in ADH. Each dot represents an individual ADH lesion.

We also asked whether in these ADH samples, the rates of pSTAT5 or pSTAT3 were inversely correlated with cell apoptosis. Apoptosis was previously found to be low (0.2–0.3%) in ADH [47, 48]. We found that in our ADH samples (n = 59), the apoptosis rate was also low, ranging from 0 to 2.34% with the medium value at approximately 0.16%. Similar lower rates of apoptosis were also found in normal breast tissues (n = 31) and UDH cases (n = 15) (Fig 4A). There was no significant association between the rates of pSTAT5-postive cells or pSTAT3-positive cells and cell apoptosis (Fig 4B and 4C).

## Discussion

In this study of pSTAT5 and pSTAT3 status in human precancerous lesions, we found that both STAT5 and STAT3 were activated in significant percentages of cells in ADH (Fig 1A–1F). While the frequency of pSTAT5-postive cells in these lesions was similar to that in normal breast epithelia (Fig 1B), the frequency of pSTAT3-postive cells was modestly higher than that in normal breast epithelia (Fig 1D). We have previously reported preclinical evidence that blockade of STAT5 activity can prevent breast cancer [24]. Therefore, the observations in this study suggest that prophylactic therapy targeting STAT5 and STAT3 in high-risk women may also lower their breast cancer risk.

STAT5 and STAT3 are activated at different stages of mammary gland development and play distinct and reciprocal roles, but in a significant proportions of breast cancers, both STAT5 and STAT3 are activated [26, 49]. It is unclear when in breast cancer evolution these two proteins become concordantly activated. We found that the expression patterns of pSTAT5 and pSTAT3 remain complementary or reciprocal in the majorities of ADH (Fig 2A and 2B). These findings suggest that the concordant regulation of STAT5 and STAT3 activities is gained at a later stage of tumor evolution. Perhaps factors that enable this concordant activation are important in driving the progression of ADH to DCIS.

In contrast to DCIS and IDC, cell proliferation in ADH is generally very low (Fig 3A), suggesting that they are not rapidly advancing. This observation may not be surprising since the majority of precancerous lesions do not advance and may even regress, while the rapidly advancing ones transition through this stage quickly and may thus be difficult to capture in this type of sampling. Consequently, the status of pSTAT5 and pSTAT3 in the rapidly progressing lesions is difficult to assess in a clinical setting. It is plausible that pSTAT5 and pSTAT3 play key roles in driving the progression from ADH to DCIS and IBC.

Apoptosis was also found to be low in the ADH cases studied here (Fig 4A). This may be predicted based on low levels of proliferation in these lesions: apoptosis is often activated in response to potent oncogenic signaling and to aberrant cell proliferation-induced DNA replicative stress [50, 51], but lack of potent proliferation in these lesions is indicative of weak oncogenic signaling and is not predicted to cause DNA replicative stress. Consequently, most of these lesions are not under strong evolutionary pressure to activate STAT5 and/or STAT3 to block apoptosis, unlike what we observed in mouse models in which the early lesions are under stimulation of potent oncogenic signaling [24]. Therefore, it is not surprising that the low apoptosis rates in these human ADHs are not associated with pSTAT5 or pSTAT3.

In conclusion, we have determined the status of pSTAT5 and pSTAT3 in a relatively small number of human ADH cases, and detected a reciprocal and complementary expression pattern of pSTAT5 and pSTAT3 in these lesions, suggesting that these lesions may be closer to normal breast epithelia than to DCIS or IBC in terms of regulation of STAT5 and STAT3 activities. It is of interest to note that these samples were from Chinese women, who are generally low in breast cancer risk relative to White, Hispanic, and African-American women [52–54]; it remains to be determined whether findings in this report hold true in these other populations of women.

## References

[1] Bartow SA, Pathak DR, Black WC, Key CR, Teaf SR. Prevalence of benign, atypical, and malignant breast lesions in populations at different risk for breast cancer. A forensic autopsy study. Cancer. 1987;60(11):2751–60. Epub 1987/12/01. . 3677009 10.1002/1097-0142(19871201)60:11<2751::aid-cncr2820601127>3.0.co;2-m

[2] Nielsen M, Jensen J, Andersen J. Precancerous and cancerous breast lesions during lifetime and at autopsy. A study of 83 women. Cancer. 1984;54(4):612–5. Epub 1984/08/15. . 6744199 10.1002/1097-0142(1984)54:4<612::aid-cncr2820540403>3.0.co;2-b

[3] Santen RJ, Allred DC, Ardoin SP, Archer DF, Boyd N, Braunstein GD, et al Postmenopausal hormone therapy: an Endocrine Society scientific statement. The Journal of clinical endocrinology and metabolism. 2010;95(7 Suppl 1):s1–s66. Epub 2010/06/23. 10.1210/jc.2009-2509 . 20566620 PMC6287288

[4] Wellings SR, Jensen HM, Marcum RG. An atlas of subgross pathology of the human breast with special reference to possible precancerous lesions. Journal of the National Cancer Institute. 1975;55(2):231–73. Epub 1975/08/01. . 169369

[5] Allred DC, Mohsin SK. Biological features of premalignant disease in the human breast. J Mammary Gland Biol Neoplasia. 2000;5(4):351–64. . 14973381 10.1023/a:1009573710675

[6] Page DL, Dupont WD, Rogers LW, Rados MS. Atypical hyperplastic lesions of the female breast. A long-term follow-up study. Cancer. 1985;55(11):2698–708. Epub 1985/06/01. . 2986821 10.1002/1097-0142(19850601)55:11<2698::aid-cncr2820551127>3.0.co;2-a

[7] Page DL, Dupont WD. Anatomic indicators (histologic and cytologic) of increased breast cancer risk. Breast cancer research and treatment. 1993;28(2):157–66. Epub 1993/11/01. . 8173068 10.1007/BF00666428

[8] Dupont WD, Page DL. Risk factors for breast cancer in women with proliferative breast disease. The New England journal of medicine. 1985;312(3):146–51. Epub 1985/01/17. 10.1056/NEJM198501173120303 . 3965932

[9] Hartmann LC, Degnim AC, Santen RJ, Dupont WD, Ghosh K. Atypical hyperplasia of the breast—risk assessment and management options. The New England journal of medicine. 2015;372(1):78–89. Epub 2015/01/01. 10.1056/NEJMsr1407164 . 25551530 PMC4347900

[10] Hartmann LC, Sellers TA, Frost MH, Lingle WL, Degnim AC, Ghosh K, et al Benign breast disease and the risk of breast cancer. The New England journal of medicine. 2005;353(3):229–37. Epub 2005/07/22. 10.1056/NEJMoa044383 . 16034008

[11] Ma XJ, Salunga R, Tuggle JT, Gaudet J, Enright E, McQuary P, et al Gene expression profiles of human breast cancer progression. Proceedings of the National Academy of Sciences of the United States of America. 2003;100(10):5974–9. ; PubMed Central PMCID: PMCfile:\\\C:\PDF\2003\Ma-sgroi.pdf. 12714683 10.1073/pnas.0931261100 PMC156311

[12] Hennighausen L, Robinson GW. Interpretation of cytokine signaling through the transcription factors STAT5A and STAT5B. Genes & development. 2008;22(6):711–21. . 18347089 10.1101/gad.1643908 PMC2394721

[13] Fahrenkamp D, de Leur HS, Kuster A, Chatain N, Muller-Newen G. Src family kinases interfere with dimerization of STAT5A through a phosphotyrosine-SH2 domain interaction. Cell communication and signaling: CCS. 2015;13(1):10 Epub 2015/04/18. 10.1186/s12964-014-0081-7 25885255 PMC4350284

[14] Haricharan S, Li Y. STAT signaling in mammary gland differentiation, cell survival and tumorigenesis. Molecular and cellular endocrinology. 2014;382(1):560–9. Epub 2013/04/02. 10.1016/j.mce.2013.03.014 23541951 PMC3748257

[15] Nevalainen MT, Xie J, Bubendorf L, Wagner KU, Rui H. Basal activation of transcription factor signal transducer and activator of transcription (Stat5) in nonpregnant mouse and human breast epithelium. Molecular endocrinology. 2002;16(5):1108–24. . 11981045 10.1210/mend.16.5.0839

[16] Liu X, Robinson GW, Hennighausen L. Activation of Stat5a and Stat5b by tyrosine phosphorylation is tightly linked to mammary gland differentiation. Molecular endocrinology. 1996;10(12):1496–506. Epub 1996/12/01. 10.1210/mend.10.12.8961260 . 8961260

[17] Cotarla I, Ren S, Zhang Y, Gehan E, Singh B, Furth PA. Stat5a is tyrosine phosphorylated and nuclear localized in a high proportion of human breast cancers. Int J Cancer. 2004;108(5):665–71. . 14696092 10.1002/ijc.11619

[18] Dong J, Tong T, Reynado AM, Rosen JM, Huang S, Li Y. Genetic manipulation of individual somatic mammary cells in vivo reveals a master role of STAT5a in inducing alveolar fate commitment and lactogenesis even in the absence of ovarian hormones. Dev Biol. 2010;346(2):196–203. Epub 2010/08/10. 10.1016/j.ydbio.2010.07.027 20691178 PMC3020144

[19] Wagner KU, Rui H. Jak2/Stat5 signaling in mammogenesis, breast cancer initiation and progression. J Mammary Gland Biol Neoplasia. 2008;13(1):93–103. 10.1007/s10911-008-9062-z 18228120

[20] Watson CJ, Neoh K. The Stat family of transcription factors have diverse roles in mammary gland development. Seminars in cell & developmental biology. 2008;19(4):401–6. Epub 2008/08/30. doi: S1084-9521(08)00054-2 [pii] 10.1016/j.semcdb.2008.07.021 . 18723104

[21] Bednorz NL, Brill B, Klein A, Gabel K, Groner B. Tracking the activation of Stat5 through the expression of an inducible reporter gene in a transgenic mouse line. Endocrinology. 2011;152(5):1935–47. 10.1210/en.2011-0053 21427222

[22] Nevalainen MT, Xie J, Torhorst J, Bubendorf L, Haas P, Kononen J, et al Signal transducer and activator of transcription-5 activation and breast cancer prognosis. Journal of clinical oncology: official journal of the American Society of Clinical Oncology. 2004;22(11):2053–60. . 15169792 10.1200/JCO.2004.11.046

[23] Peck AR, Witkiewicz AK, Liu C, Stringer GA, Klimowicz AC, Pequignot E, et al Loss of nuclear localized and tyrosine phosphorylated Stat5 in breast cancer predicts poor clinical outcome and increased risk of antiestrogen therapy failure. Journal of clinical oncology: official journal of the American Society of Clinical Oncology. 2011;29(18):2448–58. Epub 2011/05/18. 10.1200/JCO.2010.30.3552 . 21576635 PMC3675698

[24] Haricharan S, Dong J, Hein S, Reddy JP, Du Z, Toneff M, et al Mechanism and preclinical prevention of increased breast cancer risk caused by pregnancy. eLife. 2013;2:e00996 Epub 2014/01/02. 10.7554/eLife.00996 24381245 PMC3874103

[25] Santen RJ. Menopausal hormone therapy and breast cancer. The Journal of steroid biochemistry and molecular biology. 2014;142:52–61. Epub 2013/07/23. 10.1016/j.jsbmb.2013.06.010 . 23871991

[26] Walker SR, Xiang M, Frank DA. Distinct roles of STAT3 and STAT5 in the pathogenesis and targeted therapy of breast cancer. Molecular and cellular endocrinology. 2014;382(1):616–21. Epub 2013/03/28. 10.1016/j.mce.2013.03.010 23531638 PMC3732813

[27] Hughes K, Watson CJ. The spectrum of STAT functions in mammary gland development. Jak-Stat. 2012;1(3):151–8. Epub 2012/07/01. 10.4161/jkst.19691 24058764 PMC3670238

[28] Wei W, Tweardy DJ, Zhang M, Zhang X, Landua J, Petrovic I, et al STAT3 signaling is activated preferentially in tumor-initiating cells in claudin-low models of human breast cancer. Stem cells. 2014;32(10):2571–82. Epub 2014/06/04. 10.1002/stem.1752 . 24891218

[29] Marotta LL, Almendro V, Marusyk A, Shipitsin M, Schemme J, Walker SR, et al The JAK2/STAT3 signaling pathway is required for growth of CD44(+)CD24(-) stem cell-like breast cancer cells in human tumors. The Journal of clinical investigation. 2011;121(7):2723–35. Epub 2011/06/03. 10.1172/JCI44745 21633165 PMC3223826

[30] Sato T, Neilson LM, Peck AR, Liu C, Tran TH, Witkiewicz A, et al Signal transducer and activator of transcription-3 and breast cancer prognosis. American journal of cancer research. 2011;1(3):347–55. Epub 2011/07/22. 21776434 PMC3138712

[31] Diaz N, Minton S, Cox C, Bowman T, Gritsko T, Garcia R, et al Activation of stat3 in primary tumors from high-risk breast cancer patients is associated with elevated levels of activated SRC and survivin expression. Clin Cancer Res. 2006;12(1):20–8. . 16397019 10.1158/1078-0432.CCR-04-1749

[32] Watson CJ, Miller WR. Elevated levels of members of the STAT family of transcription factors in breast carcinoma nuclear extracts. British journal of cancer. 1995;71(4):840–4. . 7710952 10.1038/bjc.1995.162 PMC2033751

[33] Garcia R, Bowman TL, Niu G, Yu H, Minton S, Muro-Cacho CA, et al Constitutive activation of Stat3 by the Src and JAK tyrosine kinases participates in growth regulation of human breast carcinoma cells. Oncogene. 2001;20(20):2499–513. . 11420660 10.1038/sj.onc.1204349

[34] Charpin C, Secq V, Giusiano S, Carpentier S, Andrac L, Lavaut MN, et al A signature predictive of disease outcome in breast carcinomas, identified by quantitative immunocytochemical assays. International journal of cancer Journal international du cancer. 2009;124(9):2124–34. Epub 2009/01/15. 10.1002/ijc.24177 . 19142869

[35] Hoshi K, Tokunaga M, Mochizuki M, Ohtake T, Katagata N, Wakasa H, et al [Pathological characterization of atypical ductal hyperplasia of the breast]. Gan to kagaku ryoho Cancer & chemotherapy. 1995;22 Suppl 1:36–41. Epub 1995/04/01. . 7747990

[36] De Potter CR, Praet MM, Slavin RE, Verbeeck P, Roels HJ. Feulgen DNA content and mitotic activity in proliferative breast disease. A comparison with ductal carcinoma in situ. Histopathology. 1987;11(12):1307–19. Epub 1987/12/01. . 2831133 10.1111/j.1365-2559.1987.tb01875.x

[37] Meyer JS. Cell proliferation in normal human breast ducts, fibroadenomas, and other ductal hyperplasias measured by nuclear labeling with tritiated thymidine. Effects of menstrual phase, age, and oral contraceptive hormones. Human pathology. 1977;8(1):67–81. Epub 1977/01/01. . 844855 10.1016/s0046-8177(77)80066-x

[38] Ferguson DJ, Anderson TJ. Morphological evaluation of cell turnover in relation to the menstrual cycle in the "resting" human breast. British journal of cancer. 1981;44(2):177–81. Epub 1981/08/01. 7272186 10.1038/bjc.1981.168 PMC2010743

[39] Joshi K, Smith JA, Perusinghe N, Monoghan P. Cell proliferation in the human mammary epithelium. Differential contribution by epithelial and myoepithelial cells. The American journal of pathology. 1986;124(2):199–206. Epub 1986/08/01. 3740213 PMC1888284

[40] Longacre TA, Bartow SA. A correlative morphologic study of human breast and endometrium in the menstrual cycle. The American journal of surgical pathology. 1986;10(6):382–93. Epub 1986/06/01. . 3717495 10.1097/00000478-198606000-00003

[41] Russo J, Calaf G, Roi L, Russo IH. Influence of age and gland topography on cell kinetics of normal human breast tissue. Journal of the National Cancer Institute. 1987;78(3):413–8. Epub 1987/03/01. . 3469454

[42] Going JJ, Anderson TJ, Battersby S, MacIntyre CC. Proliferative and secretory activity in human breast during natural and artificial menstrual cycles. The American journal of pathology. 1988;130(1):193–204. Epub 1988/01/01. . 3337211 PMC1880536

[43] Potten CS, Watson RJ, Williams GT, Tickle S, Roberts SA, Harris M, et al The effect of age and menstrual cycle upon proliferative activity of the normal human breast. British journal of cancer. 1988;58(2):163–70. Epub 1988/08/01. 3166907 10.1038/bjc.1988.185 PMC2246757

[44] Kamel OW, Franklin WA, Ringus JC, Meyer JS. Thymidine labeling index and Ki-67 growth fraction in lesions of the breast. The American journal of pathology. 1989;134(1):107–13. Epub 1989/01/01. 2536520 PMC1879539

[45] Schmitt FC. Multistep progression from an oestrogen-dependent growth towards an autonomous growth in breast carcinogenesis. European journal of cancer. 1995;31A(12):2049–52. Epub 1995/11/01. . 8562164 10.1016/0959-8049(95)00430-0

[46] Visscher DW, Gingrich DS, Buckley J, Tabaczka P, Crissman JD. Cell cycle analysis of normal, atrophic, and hyperplastic breast epithelium using two-color multiparametric flow cytometry. Analytical cellular pathology: the journal of the European Society for Analytical Cellular Pathology. 1996;12(2):115–24. Epub 1996/11/01. . 8986295

[47] Vakkala M, Paakko P, Soini Y. Expression of caspases 3, 6 and 8 is increased in parallel with apoptosis and histological aggressiveness of the breast lesion. British journal of cancer. 1999;81(4):592–9. . 10574243 10.1038/sj.bjc.6690735 PMC2362889

[48] Mustonen M, Raunio H, Paakko P, Soini Y. The extent of apoptosis is inversely associated with bcl-2 expression in premalignant and malignant breast lesions. Histopathology. 1997;31(4):347–54. . 9363451 10.1046/j.1365-2559.1997.2710877.x

[49] Walker SR, Nelson EA, Zou L, Chaudhury M, Signoretti S, Richardson A, et al Reciprocal effects of STAT5 and STAT3 in breast cancer. Molecular cancer research: MCR. 2009;7(6):966–76. Epub 2009/06/06. 10.1158/1541-7786.MCR-08-0238 . 19491198

[50] Reddy JP, Peddibhotla S, Bu W, Zhao J, Haricharan S, Du YC, et al Defining the ATM-mediated barrier to tumorigenesis in somatic mammary cells following ErbB2 activation. Proceedings of the National Academy of Sciences of the United States of America. 2010;107(8):3728–33. Epub 2010/02/06. 10.1073/pnas.0910665107 20133707 PMC2840493

[51] Sinha VC, Qin L, Li Y. A p53/ARF-dependent anticancer barrier activates senescence and blocks tumorigenesis without impacting apoptosis. Molecular cancer research: MCR. 2015;13(2):231–8. Epub 2014/09/26. 10.1158/1541-7786.MCR-14-0481-T 25253740 PMC4336810

[52] Yuan JM, Yu MC, Ross RK, Gao YT, Henderson BE. Risk factors for breast cancer in Chinese women in Shanghai. Cancer research. 1988;48(7):1949–53. Epub 1988/04/01. . 3349468

[53] Wang QS, Yu MC, Henderson BE. Risk factors for breast cancer in Tianjin, People's Republic of China. National Cancer Institute monograph. 1985;69:39–42. Epub 1985/12/01. . 3834342

[54] Pike MC, Krailo MD, Henderson BE, Casagrande JT, Hoel DG. 'Hormonal' risk factors, 'breast tissue age' and the age-incidence of breast cancer. Nature. 1983;303(5920):767–70. 6866078 10.1038/303767a0

